# Supplementary figures and images for: Inactivation of lmo0946 (sif) induces the SOS response and MGEs mobilization and silences the general stress response and virulence program in Listeria monocytogenes
Source: Front Microbiol. 2024 Jan 4;14:1324062. doi: 10.3389/fmicb.2023.1324062 (PMC10794523; doi:10.3389/fmicb.2023.1324062)

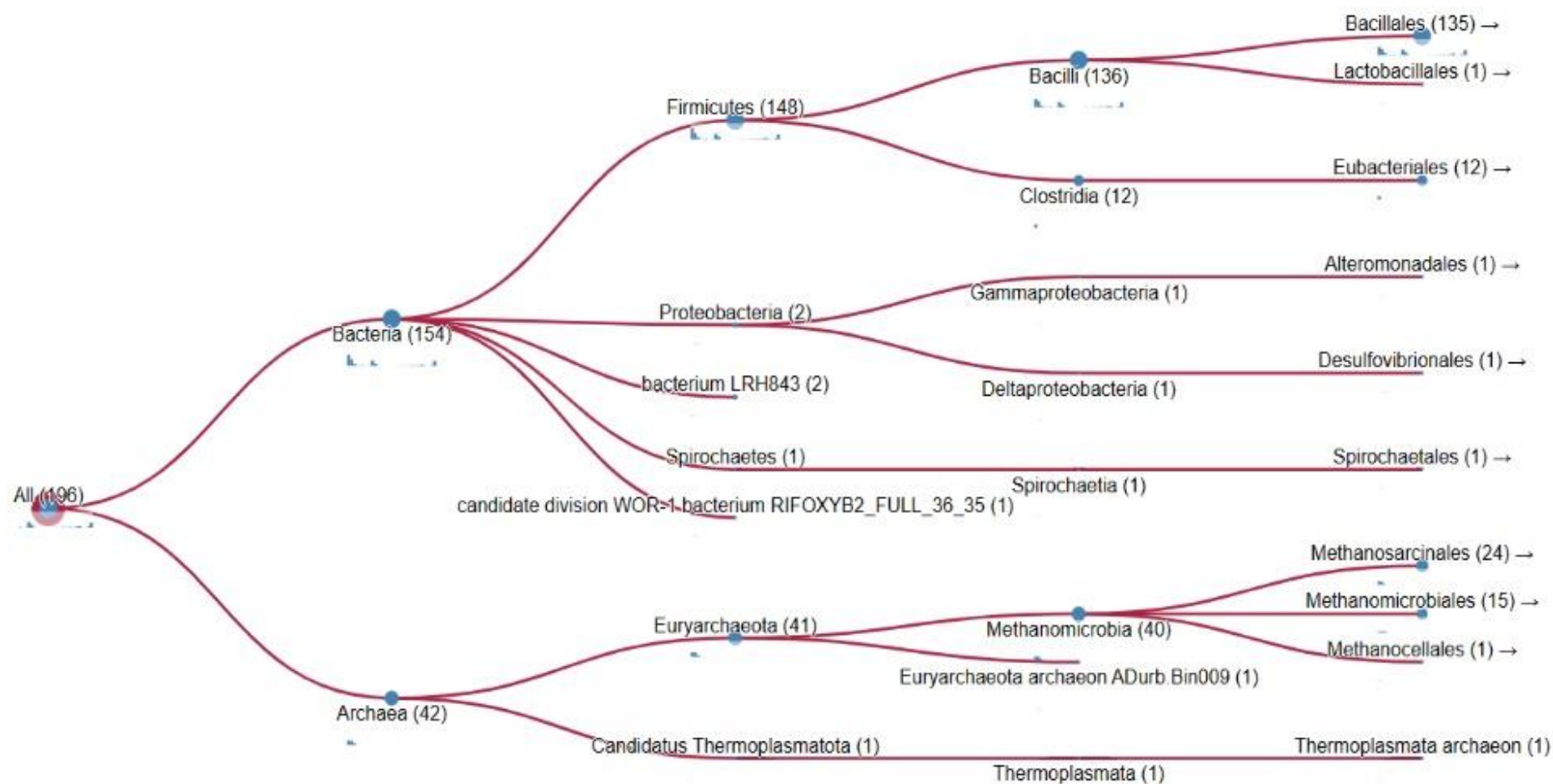

**Figure S1. PHAMMER analysis of taxonomic distribution of Lmo0946 homologs in UniProtKB database.**

Supplement: Supplementary file 1 [file Image_1.PDF]
