# Supplementary material for: Inactivation of lmo0946 (sif) induces the SOS response and MGEs mobilization and silences the general stress response and virulence program in Listeria monocytogenes
Source: Front Microbiol. 2024 Jan 4;14:1324062. doi: 10.3389/fmicb.2023.1324062 (PMC10794523; doi:10.3389/fmicb.2023.1324062)
Supplement: Supplementary file 2 [file Image_2.PDF]

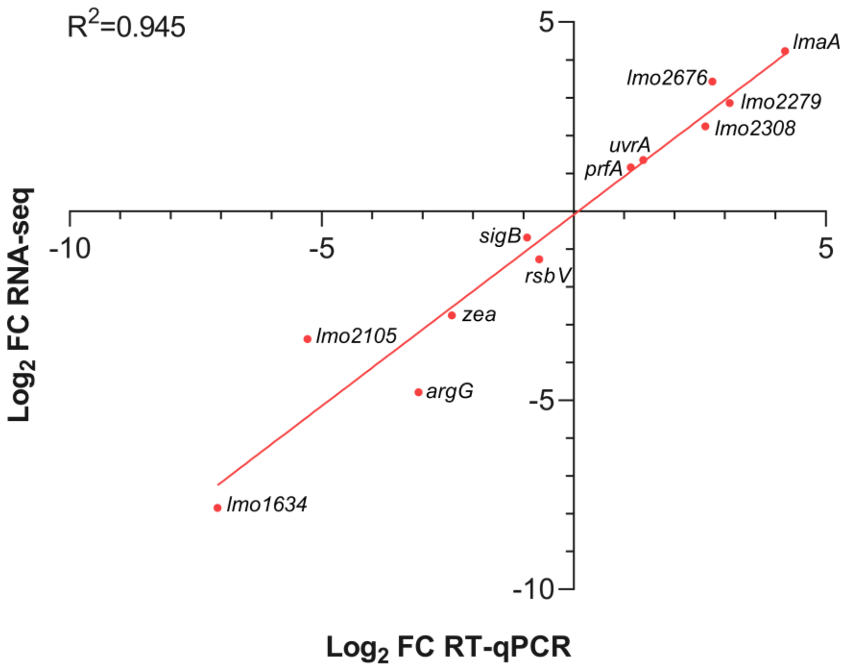

Figure S2. Validation of the RNA sequencing data by RT-qPCR analysis. Comparison of the log2 of the fold changes (FC) of 12 genes obtained in the RNA-seq and in the RT-qPCR. The data represent mean values from 3 biological replicates of experiments.
