# Supplementary material for: Inactivation of lmo0946 (sif) induces the SOS response and MGEs mobilization and silences the general stress response and virulence program in Listeria monocytogenes
Source: Front Microbiol. 2024 Jan 4;14:1324062. doi: 10.3389/fmicb.2023.1324062 (PMC10794523; doi:10.3389/fmicb.2023.1324062)
Supplement: Supplementary file 4 [file Table_1.pdf]

Supplementary Table S1: Primers used in this study

Name	Sequence (5'→ 3')	Further information
**Mutants construction**		
**DL0944FA**	ATGGATCCTGCGAGCGTAGAAGAAGCG	Forward primer for upstream flanking region of *lmo0944*. EcoRI restriction enzyme site (underlined).
**DL0944RB**	TCATGCTTGGTCCGTTACCTC	Reverse primer for upstream flanking region of *lmo0944*.
**DL0944FC**	**GTAACGGACCAAGCATGA**AGCAGCTGTAGCTGAGAAG	Forward primer for downstream flanking region of *lmo0944*. Anneals with DL0944RB (bold).
**DL0944RD**	TGTCCATGGTCTGCGTGCGGGTGTG	Reverse primer for downstream flanking region of *lmo0944*. NcoI restriction enzyme site (underlined).
**DL0945FA**	GTGGATCCTGTGCGGGATTAATCGTCTC	Forward primer for upstream flanking region of *lmo0945*. EcoRI restriction enzyme site (underlined).
**DL0945RB**	CTATGTCATCTTTCTCCTCCTGC	Reverse primer for upstream flanking region of *lmo0945*.
**DL0945FC**	**AGGAGAAAGATGACATAG**ACGCGGCGAAAAAGCGA	Forward primer for downstream flanking region of *lmo0945*. Anneals with DL0945RB (bold).
**DL0945RD**	GTGCCATGGTCATCTATGGACTGATATGGT	Reverse primer for downstream flanking region of *lmo0945*. NcoI restriction enzyme site (underlined).
**Fri Fpm**	GTGGATCCGCTTGGGCCTCCTAGTTG	Forward primer for region of *fri*. EcoRI restriction enzyme site (underlined).
**Fri Rpm**	GTTCCATGGCTTGGTCCGTTACCTCG	Reverse primer for region of *fri*. NcoI restriction enzyme site (underlined).
**Fri F sub**	CAAGTAGCGAATT**AG**AACGTATTCACA	Forward primer for site-directed mutagenesis of *fri*. Substituted nucleotides (bold).
**Fri R sub**	TGTGAATACGTT**CT**AATTCGCTACTTG	Reverse primer for site-directed mutagenesis of *fri*. Substituted nucleotides (bold).
**Fri F subsc**	GTTATCGCCGCATTTGTTCT**AG**	Forward primer for verification of chromosomal substitution in *fri*, used in pair with Fri Rpm. Substituted nucleotides (bold).
**Lmo0946 Fpm**	CAGGATCCGAAGAATTACCAGAAAGC	Forward primer for region of *lmo0946* and *lhrC5*. EcoRI restriction enzyme site (underlined).
**LhrC5 Rpm**	CTTCCATGGCACAATCTATCGGTTACT	Reverse primer for region of *lmo0946* and *lhrC5*. NcoI restriction enzyme site (underlined).

**0946 F sub**	CAGACCAAACA**TAG**TGGCAAATTGAC	Forward primer for site-directed mutagenesis of *lmo0946*. Substituted nucleotides (bold).
**0946 R sub**	GTCAATTTGCCA**CTA**TGTTTGGTCTG	Reverse primer for site-directed mutagenesis of *lmo0946*. Substituted nucleotides (bold).
**0946 F subsc**	CTATTAGAACCAGACCAAACA**T**A**G**	Forward primer for verification of chromosomal substitution in *lmo0946*, used in pair with LhrC5 Rpm. Substituted nucleotides (bold).
**LhrC5 F sub**	AGAAAAGT**C**A**G**T**GC**ATGTATAAGCTAACAACA	Forward primer for site-directed mutagenesis of *lhrC5*. Substituted nucleotides (bold).
**LhrC5 R sub**	TGTTGTTAGCTTATACAT**GC**A**C**T**G**ACTTTTCT	Reverse primer for site-directed mutagenesis of *lhrC5*. Substituted nucleotides (bold).
**LhrC5 R subsc**	GTTGTTAGCTTATACAT**GC**A**C**T**G**	Reverse primer for verification of chromosomal substitution in *lhrC5*, used in pair with Lmo0946 Fpm. Substituted nucleotides (bold).
**LhrC1-4FA**	CGAGTCGACACCGGGACCAGATGGCATG	Forward primer for upstream flanking region of *lhrC1*. SalI restriction enzyme site (underlined).
**LhrC1-4RB**	CCAAGAAAAGAAAGTTTCTGCTGTGC	Reverse primer for upstream flanking region of *lhrC1*.
**LhrC1-4FC**	**CACAGCAGAAACTTTCTTTTCTTGG**GTGCGCTAAAATC ACTAAATTCTGC	Forward primer for downstream flanking region of *lhrC4*. Anneals with LhrC1-4RB (bold).
**LhrC1-4RD**	CGAAGATCTGCTCGTCTGGTACTGATGCTGC	Reverse primer for downstream flanking region of *lhrC4*. BglII restriction enzyme site (underlined).
**Rli22FA**	AGAGTCGACGAAGTTATTGGAGGAAACATTCG	Forward primer for upstream flanking region of *rli22* (*lhrC6*). SalI restriction enzyme site (underlined).
**Rli22RB**	AAGTTCTATATTAAAGGTTAATTATGAAC	Reverse primer for upstream flanking region of *rli22* (*lhrC6*).
**Rli22FC**	**GTTCATAATTAACCTTTAATATAGAACTT**GGACCGACT TGATTGTCGG	Forward primer for downstream flanking region of *rli22* (*lhrC6*). Anneals with Rli22RB (bold).
**Rli22RD**	AGAAGATCTGCAATTAGTCCGCCAACAG	Reverse primer for downstream flanking region of *rli22* (*lhrC6*). BglII restriction enzyme site (underlined).
**Rli33-1FA**	ACAGTCGACGGACGACAACTTAGAAAAAG	Forward primer for upstream flanking region of *rli33-1* (*lhrC7*). SalI restriction enzyme site (underlined).
**Rli33-1RB**	GTTATATAATACCCGTCTCAC	Reverse primer for upstream flanking region of *rli33-1* (*lhrC7*)).
**Rli33-1FC**	**GTGAGACGGGTATTATATAAC**GGAAAAAATGCTAAATA TAAGAAAACTCC	Forward primer for downstream flanking region of *rli33-1* (*lhrC7*). Anneals with Rli33-1RB (bold).

**Rli33-1RD**	ACAAGATCTTGGTAATAACATACACTGCTAAAAC	Reverse primer for downstream flanking region of *rli33-1* (*lhrC7*). BglII restriction enzyme site (underlined).
**Lmo0946 Fcom**	CTTCTGCAGCATTGAAGATCATGATTATTGGTATTTC	Forward primer for complementation of *lmo0946**. PstI restriction enzyme site (underlined).
**Lmo0946 Rcom**	CTTGGTACCGGGAGTTTGGGATTTCCATTC	Reverse primer for complementation of *lmo0946**. KpnI restriction enzyme site (underlined).
	**RT-PCR**	
**RTLhrC5**	AGGGAGTAAACCGCACTAG	Primer used for reverse transcription in cotranscription analysis
**0943F**	CATTGGTATATGAGAGGCCAC	Forward primer used for PCR of *fri* in cotranscription analysis
**0943R**	CATTGTCGCCTTCTTTGTCAG	Reverse primer used for PCR of *fri* in cotranscription analysis
**0944F**	ATGGTTTCATGATGAGTTTGATGT	Forward primer used for PCR of *lmo0944* in cotranscription analysis
**0944R**	TCCGTTTTTGGTTCATAGTCG	Reverse primer used for PCR of *lmo0944* in cotranscription analysis
**lmaA F**	CAAGGTCTAACTGTAAACCGTTCT	Forward primer used for RT-qPCR of *lmaA*
**lmaA R**	CCAATTCTTTGTGAGACTCTGCATC	Reverse primer used for RT-qPCR of *lmaA*
**lmo2676 F**	GCCAAACTCGCGCTCGATAATG	Forward primer used for RT-qPCR of *lmo2676*
**lmo2676 R**	GAACAGCAGTCCGACGTCCAATT	Reverse primer used for RT-qPCR of *lmo2676*
**lmo2308 F**	CTGAGGTAGTTGCTGAATCAGTTCAATT	Forward primer used for RT-qPCR of *lmo2308*
**lmo2308 R**	ATCGCTCTTCTGACTCGTATCCGC	Reverse primer used for RT-qPCR of *lmo2308*
**sigB F**	ACTTCAAAGCTCGCCGCAAATTAG	Forward primer used for RT-qPCR of *sigB*
**sigB R**	ATCGTACTTCCATCCGAATCAGCTT	Reverse primer used for RT-qPCR of *sigB*
**rsbV F**	GACATATTTGTTGCTGGGGAGATCG	Forward primer used for RT-qPCR of *rsbV*
**rsbV R**	TACAAATACGCCTAATCCGGTGCTATC	Reverse primer used for RT-qPCR of *rsbV*
**lmo1634 F**	ATCAGTAGAGTGAATAACTGCGG	Forward primer used for RT-qPCR of *lmo1634*
**lmo1634 R**	GTGTTGGCGACAAATACCCA	Reverse primer used for RT-qPCR of *lmo1634*
**int F**	GCAATGGATAGAGCAATCGTTTTAGG	Forward primer used for RT-qPCR of *int*
**int R**	CTTCACCGATTCTCATGCCTGTC	Reverse primer used for RT-qPCR of *int*
**uvrA F**	TGGAAACACGCTTATTGTCGTTGAG	Forward primer used for RT-qPCR of *uvrA*
**uvrA R**	ACGTTTAGCAGGGACTGGAA	Reverse primer used for RT-qPCR of *uvrA*
**prfA F**	GCAGGCTACCGCATACGTTATC	Forward primer used for RT-qPCR of *prfA*
**prfA R**	TTCTTTACCATACACATAGGTCAGGA	Reverse primer used for RT-qPCR of *prfA*
**zea F**	GGGAATATAGCTCAAGAGAAAGAAATTG	Forward primer used for RT-qPCR of *zea*
**zea R**	ACCCTTACTAACATTAATCTTAGCATTAAACGA	Reverse primer used for RT-qPCR of *zea*
**hfq F**	GGTGGACAAGGGTTACAGGA	Forward primer used for RT-qPCR of *hfq*

**hfq R**	ACAACGCGTCCTCTTAACTGA	Reverse primer used for RT-qPCR of *hfq*
**lmo2105 F**	GCTCGTCTTTGTTCCTCAAATCTTG	Forward primer used for RT-qPCR of *lmo2105*
**lmo2105 R**	CAATCGACCGAGCTGCCATAATTC	Reverse primer used for RT-qPCR of *lmo2105*
**argG F**	TCTCCTGTCCGTGATTGGAAATG	Forward primer used for RT-qPCR of *agrG*
**argG R**	GCACACCACATTCATTACTTCTAC	Reverse primer used for RT-qPCR of *agrG*
**lmo2279 F**	CCGCCTAAGTGGCTTCCGAC	Forward primer used for RT-qPCR of *lmo2279*
**lmo2279 R**	TTGCAAGCGATCCAGAGCCG	Reverse primer used for RT-qPCR of *lmo2279*
**recA F**	GCTCATGTTGGATTACAAGCACG	Forward primer used for RT-qPCR of *recA*
**recA R**	ACGTACAGTAGAATAGAATTTAAGCGCA	Reverse primer used for RT-qPCR of *recA*
**hly F**	CCTCCTGCATATATCTCAAGTGTG	Forward primer used for RT-qPCR of *hly*
**hly R**	GAACCTCCGTAAATTACGGCTTTGA	Reverse primer used for RT-qPCR of *hly*
**rpoB F**	CGTCGTCTTCGTTCTGTTGG	Forward primer used for RT-qPCR of *rpoB*
**rpoB R**	GTTCACGAACCACACGTTCC	Reverse primer used for RT-qPCR of *rpoB*
**Northern blot analysis**		
**Fri NB F**	GGCGAACAAATGGATGAAGTA	Forward primer for double stranded probe for *fri* mRNA
**Fri NB R**	CAATACCTTGTTGATATTCGTC	Reverse primer for double stranded probe for *fri* mRNA
**lmo0944 NB F**	GGTAATGGAATTCGACTTTTTGC	Forward primer for double stranded probe for *lmo0944* mRNA
**lmo0944 NB R**	GGTTCATAGTCGATTTTCCAG	Reverse primer for double stranded probe for *lmo0944* mRNA
**lmo0945 NB F**	CCAAGACGCGGCGAAAAAGC	Forward primer for double stranded probe for *lmo0945* mRNA
**lmo0945 NB R**	CGCTTCTAAATTGGCACCGC	Reverse primer for double stranded probe for *lmo0945* mRNA
**lmo0946 NB F**	ATGAAAAAAGCAATTTTAGATCGG	Forward primer for double stranded probe for *sif* mRNA
**lmo0946 NB R**	GATGAATCGAGCTTTTCTTTG	Reverse primer for double stranded probe for *sif* mRNA
**lhrC5 NB F**	ATAAGCTAACAACAAGCAAAACATTTTCATTTCTTTCCC TTTTTA**GAATGGAAATCCCAAACTCCC**	Forward primer for double stranded probe for *lhrC5* mRNA. Anneals with lhrC5 NB R (bold).
**lhrC5 NB R**	AAAAAAACTAGTGCGGAAAAAGGGAGTAAACCGCACTA GCTAAAA**GGGAGTTTGGGATTTCCATTC**	Reverse primer for double stranded probe for *lhrC5* mRNA. Anneals with lhrC5 NB F (bold).
**MGE analysis**		
**A118 R 1**	TGTATCACTTGAACGCTTTGAC	Reverse primer no 1 for A118 mobilization analysis
**A118 F 2**	TTAGCTGATTTAGCAACAGTTGAT	Forward primer no 2 for A118 mobilization analysis
**A118 F 3**	ATG AAA AAA GAA CAA ATC AGT ACT CAG	Forward primer no 3 for A118 mobilization analysis
**A118 R 4**	TTA TTG CTC GGG ATC TTG AGG AT	Reverse primer no 4 for A118 mobilization analysis
**ICELm1 R 1**	GAATTATTTCCTTTCAACCCGCTGAGC	Reverse primer no 1 for ICElm1 mobilization analysis
**ICELm1 F 2**	GCTGTAGGTTCATTCAACACAAGA	Forward primer no 2 for ICElm1 mobilization analysis

**ICELm1 F 3**	GTTATGGGTGATGGTAGAACGTATGACC	Forward primer no 3 for ICElm1 mobilization analysis
**ICELm1 R 4**	AGTCGAGTATGCGTACTTCTTCATCAA G	Reverse primer no 4 for ICElm1 mobilization analysis
**EMSA analysis**		
**Sif F_pET28a**	GAACATATGAAAAAAGCAATTTTAGATCGGATAGAAG	Forward primer, cloning of *sif* to pET28a. NdeI restriction enzyme site (underlined)
**Sif R_pET28a**	TGAGCTCAGTTTCTCCCTCTTAATCTTTCTAG	Reverse primer, cloning of *sif* to pET28a. SacI restriction enzyme site (underlined)
***hfq*_F_EMSA**	CGCGAAAAGACAGTTAACGTGGT	Forward primer for EMSA DNA of *hfq*
***hfq*_R_EMSA**	GCGACATTCTTCTGCGGGG	Reverse primer for for EMSA DNA of *hfq*
***cadA*_F_EMSA**	CTGGTCTGTAAGGGTAGCAC	Forward primer for EMSA DNA of *cadA*
***cadA*_R_EMSA**	TGCATCAGTTACACCCTCAAG	Reverse primer for for EMSA DNA of *cadA*
***argC*_F_EMSA**	GTAGCGGAAACTGCGCAAGTG	Forward primer for EMSA DNA of *argC*
***argC*_R_EMSA**	AGCGAATTAGCTCAAGTCCTC	Reverse primer for for EMSA DNA of *argC*
***lap*_F_EMSA**	TGCTACTTACTCTTCTTCAATGCT	Forward primer for EMSA DNA of *lap*
***lap*_R_EMSA**	TGGTTGAAAAGTCCTTTTGTAAAGT	Reverse primer for for EMSA DNA of *lap*
***prfA*_F_EMSA**	CTGCGCGTTGATGACAAAGG	Forward primer for EMSA DNA of *prfA*
***prfA*_R_EMSA**	GCGGTATGTTTCCACTGTCG	Reverse primer for for EMSA DNA of *prfA*
***recA* F EMSA**	CCGTAAAACAAGGCTTTCAGT	Forward primer for EMSA DNA of *recA*
***recA* R EMSA**	ATCCGCCAACTCCTAAAGCA	Reverse primer for for EMSA DNA of *recA*
***lmo1097* F EMSA**	AGTTTGTCTATCAAAGCCCACAAG	Forward primer for EMSA DNA of *lmo1097*
***lmo1097* R EMSA**	TCTTGTGCAAAAACTAATCAGACC	Reverse primer for for EMSA DNA of *lmo1097*
***16sRNA* F EMSA**	GTGCATTAGCTAGTTGGTAG	Forward primer for EMSA DNA of *16sRNA*
***16sRNA* R EMSA**	CAACAGTACTTTACGATCCG	Reverse primer for for EMSA DNA of *16sRNA*
