# Supplementary material for: Inactivation of lmo0946 (sif) induces the SOS response and MGEs mobilization and silences the general stress response and virulence program in Listeria monocytogenes
Source: Front Microbiol. 2024 Jan 4;14:1324062. doi: 10.3389/fmicb.2023.1324062 (PMC10794523; doi:10.3389/fmicb.2023.1324062)
Supplement: Supplementary file 5 [file Table_2.pdf]

Supplementary Table S2. Generation time of *L.monocytogenes* strains in different conditions of growth on the basis of exponential phase of growth analysis.

	**Generation time [minutes] ± SD**[a]			**Statistical significance [adjusted *P* value]**[b]		
Strain / Conditions of growth	EGD-e	*lmo0946**	*lmo0946*- lmo0946*	EGD-e vs. *lmo0946**	EGD-e vs. *lmo0946*-lmo0946*	lmo0946* vs. *lmo0946*-lmo0946*
37 °C, BHI (stress-free)	45.0 ± 0.7	49.9 ± 1.1	44.3 ± 1.7	0.0068 **	0.7524 ns	0.0034 **
Ethanol 5 %	93.6 ± 2.7	104.4 ± 1.8	86.4 ± 6.0	0.0368 *	0.1418 ns	0,0034 **
Penicillin G 0.09 µg/ml	54.0 ± 0.3	61.7 ± 1.0	53.9 ± 0.2	<0.0001 ****	0.9892 ns	<0.0001 ****
pH 5	73.4 ± 1.0	89.9 ± 2.0	78.3 ± 3.2	0.0003 ***	0.0873 ns	0.0019 **
pH 9	65.1 ± 1.7	72.5 ± 0.9	61.1 ± 0.6	0.0007 ***	0.0149 *	<0.0001 ****

[a] The results were calculated on the basis of three independent experiments; ± the standard deviation (SD).

[b] ANOVA with a Tukey's multiple comparisons posttest was used to determine statistical significances; The asterisks indicate a significant differences (*$P < 0.05$, **$P < 0.01$, ***$P < 0.001$, ****$P < 0.0001$); ns, non-significant.
