# Supplementary material for: Inactivation of lmo0946 (sif) induces the SOS response and MGEs mobilization and silences the general stress response and virulence program in Listeria monocytogenes
Source: Front Microbiol. 2024 Jan 4;14:1324062. doi: 10.3389/fmicb.2023.1324062 (PMC10794523; doi:10.3389/fmicb.2023.1324062)
Supplement: Supplementary file 6 [file Table_3.pdf]

Supplementary Table S3. Antibiotics and cadmium susceptibility of wild-type *L. monocytogenes* EGD-e and *L. monocytogenes lmo0946** strains

**Compound**[a]	**Strain**			
	**EGD-e**		***lmo0946****	
	Zone of inhibition (mm) ± SD[b]	MIC (µg/mL)[c]	Zone of inhibition (mm) ± SD[b]	MIC (µg/mL)[c]
Penicillin G (10)	29.2 ± 1.0	0.12	31.0 ± 0.6 [ns]	0.12
Ampicillin (10)	34.9 ± 0.9	ND	36.8 ± 0.8 [ns]	ND
Cefuroxime (30)	19.6 ± 0.8	8	27.3 ± 1.0 ***	4
Cefoxitin (30)	17.6 ± 0.6	32	21.6 ± 1.1 **	16
Vancomycin (30)	21.8 ± 0.8	ND	23.0 ± 0.6 [ns]	ND
Aztreonam (30)	0.0 ± 0.0	ND	0.0 ± 0.0 [ns]	ND
Meropenem (10)	42.7 ± 0.8	ND	41.5 ± 1.8 [ns]	ND
Tetracycline (30)	35.2 ± 1.0	ND	36.6 ± 0.6 [ns]	ND
Rifampicin (5)	35.6 ±0.6	ND	37.0 ± 1.0 [ns]	ND
Gentamicin (10)	25 ± 0.2	ND	24.4 ± 0.6 [ns]	ND
Trimethoprim (5)	38.6 ± 1.0	ND	39.3 ± 0.6 [ns]	ND
Ciprofloxacin (5)	25.8 ± 0.6	ND	25.2 ± 0.4 [ns]	ND
Cadmium	NA	200	NA	> 800

[a] The numbers in parentheses indicate the content of the antibiotics (in µg) in the disks.

[b] Values are averages of three independent experiments; ± the standard deviation (SD). Unpaired two-tailed *t* test was used to determine statistical significances. The asterisks indicate a significant differences (***P* < 0.01, ****P* < 0.001); ns, non-significant.

[c] ND, not determined; NA, not applicable.
