# Supplementary material for: Inactivation of lmo0946 (sif) induces the SOS response and MGEs mobilization and silences the general stress response and virulence program in Listeria monocytogenes
Source: Front Microbiol. 2024 Jan 4;14:1324062. doi: 10.3389/fmicb.2023.1324062 (PMC10794523; doi:10.3389/fmicb.2023.1324062)
Supplement: Supplementary file 9 [file Table_6.pdf]

Supplementary Table S6. Genes ≥ 4.0-fold up-regulated and ≤ - 4.0-fold down-regulated (with *P*adj < 0.01) in *L. monocytogenes lmo0946** compared to wild-type *L. monocytogenes* EGD-e strain in cultures from the exponential phase of growth in BHI in 37°C.

Gene name	Gene symbol	log_2 Fold Change	Regulation[a]	Product[b]	COG[b]
lmo1097		13.71		Integrase, superantigen-encoding pathogenicity islands SaPI	Replication, recombination and repair
lmo1295		12.65	**SigB** [1] ↑	RNA-binding protein Hfq	Transcription; Translation
lmo1100	*cadA*	12.27		Cadmium resistance protein	Inorganic ion transport and metabolism
lmo1101	*lspB*	9.12		Hypothetical protein	Cell wall/membrane biogenesis; Intracellular trafficking and secretion
lmo1102	*cadC*	7.85		Cadmium efflux system accessory protein	Transcription
lmo1105		6.36		Membrane protein, putative	Not in COGs
lmo1112		5.29		Hypothetical protein	Cell cycle control, mitosis and meiosis
lmo1106		5.18		Hypothetical protein	Not in COGs
lmo1115		4.96		Similar to fibrinogen-binding protein	Cell wall/membrane biogenesis
lmo2325		4.81		Hypothetical protein	Not in COGs
lmo0119		4.61		Hypothetical protein	Not in COGs
lmo0117	*lmaB*	4.61		Antigen B	Not in COGs
lmo2294		4.53	**CodY** [7] ↑	Protein gp9	Signal transduction mechanisms
lmo0118	*lmaA*	4.52		Antigen A	Function unknown
lmo0120		4.50		Hypothetical protein	Not in COGs
lmo2321		4.35		Protein gp45 [Bacteriophage A118]	Not in COGs
lmo2289		4.35		Protein gp14	Not in COGs
lmo0126		4.32		Hypothetical protein	Not in COGs
lmo0121		4.28		Phage tail length tape-measure protein	Function unknown
lmo0125		4.26		Hypothetical protein	Not in COGs
lmo0127		4.24		Hypothetical protein	Not in COGs
lmo0122		4.23		Phage tail fiber	Not in COGs
lmo2292		4.19	**CodY** [7] ↑	Protein gp11	Not in COGs
lmo0123		4.15		Putative tail or base plate protein gp18 [Bacteriophage A118]	Not in COGs

lmo2324		4.12	**CodY** [7] ↑	Phage antirepressor protein / Antirepressor [Bacteriophage A118]	Transcription; Function unknown
lmo0129		4.12		N-acetylmuramoyl-L-alanine amidase	Cell wall/membrane biogenesis
lmo0128		3.99		Similar to phage-related protein	General function prediction only
lmo0124		3.96		Hypothetical protein	Not in COGs
lmo2315		3.90		Protein gp51 [Bacteriophage A118]	Not in COGs
lmo2316		3.88		Methyltransferase	Replication, recombination and repair
lmo2326		3.82		Protein gp41 [Bacteriophage A118]	Not in COGs
lmo2298		3.81	**CodY** [7] ↑	Protein gp4	Not in COGs
lmo2313		3.75	**CodY** [7] ↑	Hypothetical protein, Lmo2313 homolog [Bacteriophage A118]	Not in COGs
lmo0115	*lmaD*	3.74		Listeria protein LmaD, associated with virulence	Not in COGs
lmo2288		3.74	**CodY** [7] ↑	Protein gp15	Not in COGs
lmo2676	*umuC*	3.70	**LexA/RecA** [3] ↑ **CodY** [7] ↓	Hypothetical protein	Replication, recombination and repair
lmo2675	*umuD*	3.70	**LexA/RecA** [3] ↑ **CodY** [7] ↓	Hypothetical protein	Not in COGs
lmo2291		3.68	**CodY** [7] ↑	Major tail shaft protein	Not in COGs
lmo0116	*lmaC*	3.66		LmaC, associated with virulence in Listeria	Not in COGs
lmo2322		3.64		gp44	Not in COGs
lmo2296		3.62	**CodY** [7] ↑	Phage capsid protein	Not in COGs
lmo2327		3.59		Hypothetical protein	Not in COGs
lmo2282		3.59	**CodY** [7] ↑	protein gp21	Not in COGs
lmo2323		3.58		Protein gp43 [Bacteriophage A118]	Not in COGs
lmo2318		3.52	**CodY** [7] ↑	Putative recombination protein / Single-stranded DNA-binding protein	Not in COGs
lmo2271		3.51	**LexA/RecA** [3] ↑	Hypothetical protein	Not in COGs
lmo2285		3.51	**CodY** [7] ↑	Protein gp18	Translation
rli38		3.51			
lmo2319		3.50		Hypothetical protein	Not in COGs
lmo2299		3.46	**CodY** [7] ↑	Putative portal protein	Not in COGs
lmo2293		3.39	**CodY** [7] ↑	Protein gp10	Not in COGs

lmo2317		3.35	**CodY** [7] ↑	Protein gp49, replication initiation [Bacteriophage A118]	Replication, recombination and repair
lmo2300		3.33	**CodY** [7] ↑	Putative terminase large subunit from bacteriophage A118	General function prediction only
lmo2295		3.30	**CodY** [7] ↑	Protein gp8	Not in COGs
lmo2297		3.29	**CodY** [7] ↑	Putative scaffolding protein	Cell motility; Signal transduction mechanisms
lmo2290		3.24	**CodY** [7] ↑	Protein gp13	Cell motility
lmo2287		3.20	**CodY** [7] ↑	Putative tape-measure	Not in COGs
lmo0152		3.20	**VirRS** [8] ↑	Oligopeptide ABC transporter, periplasmic oligopeptide-binding protein	Amino acid transport and metabolism
lmo2301		3.14	**CodY** [7] ↑	Terminase small subunit [Bacteriophage A118]	Replication, recombination and repair
rli140		3.14			
lmo2278	*lysA*	3.09	**CodY** [7] ↑	L-alanoyl-D-glutamate peptidase	Not in COGs
lmo2284		3.09	**CodY** [7] ↑	Protein gp19	Not in COGs
lmo2303		3.06	**CodY** [7] ↑	Protein gp66	Not in COGs
lmo2320		2.88		Hypothetical protein	Not in COGs
lmo2283		2.84	**CodY** [7] ↑	Protein gp20	Not in COGs
lmo2305		2.80	**CodY** [7] ↑	Hypothetical protein, Lmo2305 homolog [Bacteriophage A118]	Not in COGs
lmo2312		2.79		Conserved hypothetical protein	Function unknown
lmo2279		2.78	**CodY** [7] ↑	Holin	Not in COGs
lmo2286		2.75	**CodY** [7] ↑	Protein gp17	Not in COGs
lmo0135		2.65	**VirRS** [8] ↑	Oligopeptide ABC transporter, periplasmic oligopeptide-binding protein	Amino acid transport and metabolism
lmo2352		2.63		HTH-type transcriptional regulator YtlI, LysR family	Transcription
lmo2828		2.58	**LexA/RecA** [3] ↑ **CodY** [7] ↓	Hypothetical protein	Not in COGs
lmo2311		2.46		Hypothetical protein	Not in COGs
lmo2280		2.45	**CodY** [7] ↑	Protein gp23	Not in COGs
lmo2308		2.34		Single-stranded DNA-binding protein (prophage associated)	Replication, recombination and repair
lmo2306		2.34	**CodY** [7] ↑	Hypothetical protein	Not in COGs
lmo1975	*dinB*	2.29	**LexA/RecA** [3] ↑	DNA polymerase IV	Replication, recombination and repair
lmo2744		2.23	**CodY** [7] ↓	Cyclic nucleotide-binding protein	Signal transduction mechanisms

lmo0136		2.18	**VirRS** [8] ↑	Oligopeptide transport system permease protein	Amino acid transport and metabolism; Inorganic ion transport and metabolism
lmo2433	*estA*	2.16	**VirRS** [8] ↑	Putative esterase	General function prediction only
lmo2314		2.09		Hypothetical protein	Not in COGs
lmo2568		2.06	**CodY** [7] ↑	Hypothetical protein	Not in COGs
lmo2210		2.04	**LisR** [5] ↑	Hypothetical protein	Not in COGs
lmo1634	*lap*	-7.69		Bifunctional acetaldehyde-CoA/alcohol dehydrogenase	Energy production and conversion
lmo1591	*argC*	-4.96	**CodY** [7] ↓	N-acetyl-gamma-glutamyl-phosphate reductase	Amino acid transport and metabolism
lmo2090	*argG*	-4.93	**CodY** [7] ↓	Argininosuccinate synthase	Amino acid transport and metabolism
lmo2172		-4.28		Acetyl-CoA:acetoacetyl-CoA transferase, alpha subunit	Lipid transport and metabolism
lmo1590	*argJ*	-4.13	**CodY** [7] ↓	bifunctional ornithine acetyltransferase/N-acetylglutamate synthase protein	Amino acid transport and metabolism
lmo1257		-4.04		Hypothetical protein	Not in COGs
lmo2235		-3.91		Similar to NADH oxidase	Energy production and conversion; General function prediction only
lmo2158		-3.59	**SigB** [1] ↑ **CodY** [7] ↑	hypothetical protein	Function unknown
lmo2104	*feoA*	-3.55	**Fur** [4] ↓	Hypothetical protein	Inorganic ion transport and metabolism
lmo2091	*argH*	-3.53	**CodY** [7] ↓	argininosuccinate lyase	Amino acid transport and metabolism
lmo2234		-3.52	**CodY** [7] ↓	Inosose isomerase	Carbohydrate transport and metabolism
lmo2250	*arpJ*	-3.46	**VirRS** [8] ↑ **CodY** [7] ↓	Amino acid ABC transporter, amino acid-binding/permease protein	Amino acid transport and metabolism; Signal transduction mechanisms
lmo2410		-3.45		Hypothetical protein	Not in COGs
lmo2105	*feoB*	-3.10	**Fur** [4] ↓	Ferrous iron transport protein B	Inorganic ion transport and metabolism
rli119		-2.79			
lmo0412		-2.78		Hypothetical protein	Not in COGs
lmo0654		-2.68	**PrfA** [2] ↑; **CodY** [7] ↑	Hypothetical protein	Not in COGs
lmo2236		-2.65		Shikimate 5-dehydrogenase	Amino acid transport and metabolism
lmo2173		-2.61	**CodY** [7] ↑	Sigma-54 dependent transcriptional regulator	Transcription; Signal transduction mechanisms
lmo1406	*pflB*	-2.60	**CodY** [7] ↑	pyruvate formate-lyase	Energy production and conversion
lmo2251		-2.59	**VirRS** [8] ↑	Amino acid ABC transporter, ATP-binding protein	Amino acid transport and metabolism
lmo2686	*zea*	-2.55		Hypothetical protein	Not in COGs

lmo1917	*pflA*	-2.55		Pyruvate formate-lyase	Energy production and conversion
lmo2238		-2.55		Major facilitator family transporter	Carbohydrate transport and metabolism; Amino acid transport and metabolism; Inorganic ion transport and metabolism; General function prediction only
lmo2637	*pplA*	-2.50		Putative pheromone precursor lipoprotein, related to Cad	Function unknown
lmo0321		-2.48	**CodY [7] ↑**	membrane protein	Not in COGs
lmo0912		-2.46	**CodY [7] ↓**	Formate efflux transporter	Inorganic ion transport and metabolism
lmo0134		-2.40	**SigB [1] ↑; PrfA [2] ↑**	acetyltransferase, GNAT family	General function prediction only
lmo0628		-2.40	**CodY [7] ↑**	Hypothetical protein	Not in COGs
lmo0937		-2.37	**PrfA [2] ↑**	Hypothetical protein	Not in COGs
lmo0515		-2.37	**SigB [1] ↑**	hypothetical protein	Signal transduction mechanisms
lmo0133		-2.35	**PrfA [2] ↑**	hypothetical protein	Function unknown
lmo2171		-2.30		Oxalate/formate antiporter	Carbohydrate transport and metabolism; Amino acid transport and metabolism; Inorganic ion transport and metabolism; General function prediction only;
lmo0788		-2.28	**PrfA [2] ↑**	Activator of (R)-2-hydroxyglutaryl-CoA dehydratase	Lipid transport and metabolism; Function unknown
lmo2067	*bsh*	-2.23	**SigB [1] ↑; PrfA [2] ↑**	Choloylglycine hydrolase	Defense/virulence mechanisms
lmo1131	*cydC*	-2.22	**Fur [4] ↓**	Transport ATP-binding protein CydC	Energy production and conversion; Posttranslational modification, protein turnover, chaperones
lmo2365		-2.22		Listeria RofA-like transcriptional regulator	Transcription; Carbohydrate transport and metabolism
lmo1407	*pflC*	-2.15		pyruvate-formate lyase activating enzyme	Posttranslational modification, protein turnover, chaperones
lmo0302		-2.13		hypothetical protein	Not in COGs
lmo2434	*gadD*	-2.09	**CodY [7] ↑**	Glutamate decarboxylase	Amino acid transport and metabolism
lmo2447		-2.08		transcriptional activator	Transcription
rli105		-2.08			
lmo0953		-2.07	**SigB [1] ↑; PrfA [2] ↑; CodY [7] ↑**	hypothetical protein	Not in COGs
lmo0309		-2.04		conserved hypothetical protein	Function unknown
lmo2669		-2.02		membrane protein	Function unknown

[a] Positive regulation (↑) represents higher transcript levels in the parent strain compared to the mutant strain in regulatory gene, whereas negative regulation (↓) represent higher transcript levels in the mutant strain in regulatory gene compared to the parent strain. The data are according to:

[1] Hain et al., 2008

[2] Milohanic et al., 2003

[3] van der Veen et al., 2010
[4] McLaughlin et al., 2012
[5] Nielsen et al., 2012
[6] Hu et al., 2007
[7] Bennett et al., 2007
[8] Mandin et al., 2005
[9] Rea et al., 2005
[b] Information from Listeriomics website (listeriomics.pasteur.fr)

References:

Bennett, H.J., Pearce, D.M., Glenn, S., Taylor, C.M., Kuhn, M., Sonenshein, A.L., et al. (2007). Characterization of relA and codY mutants of *Listeria monocytogenes*: identification of the CodY regulon and its role in virulence. *Mol Microbiol* 63(5)**,** 1453-1467. doi: 10.1111/j.1365-2958.2007.05597.x.

Hain, T., Hossain, H., Chatterjee, S.S., Machata, S., Volk, U., Wagner, S., et al. (2008). Temporal transcriptomic analysis of the *Listeria monocytogenes* EGD-e sigmaB regulon. *BMC Microbiol* 8**,** 20. doi: 10.1186/1471-2180-8-20.

Hu, Y., Raengpradub, S., Schwab, U., Loss, C., Orsi, R.H., Wiedmann, M., et al. (2007). Phenotypic and transcriptomic analyses demonstrate interactions between the transcriptional regulators CtsR and Sigma B in *Listeria monocytogenes*. *Appl Environ Microbiol* 73(24)**,** 7967-7980. doi: 10.1128/AEM.01085-07.

Mandin, P., Fsihi, H., Dussurget, O., Vergassola, M., Milohanic, E., Toledo-Arana, A., et al. (2005). VirR, a response regulator critical for *Listeria monocytogenes* virulence. *Mol Microbiol* 57(5)**,** 1367-1380. doi: 10.1111/j.1365-2958.2005.04776.x.

McLaughlin, H.P., Xiao, Q., Rea, R.B., Pi, H., Casey, P.G., Darby, T., et al. (2012). A putative P-type ATPase required for virulence and resistance to haem toxicity in *Listeria monocytogenes*. *PLoS One* 7(2)**,** e30928. doi: 10.1371/journal.pone.0030928.

Milohanic, E., Glaser, P., Coppee, J.Y., Frangeul, L., Vega, Y., Vazquez-Boland, J.A., et al. (2003). Transcriptome analysis of *Listeria monocytogenes* identifies three groups of genes differently regulated by PrfA. *Mol Microbiol* 47(6)**,** 1613-1625.

Nielsen, P.K., Andersen, A.Z., Mols, M., van der Veen, S., Abee, T., and Kallipolitis, B.H. (2012). Genome-wide transcriptional profiling of the cell envelope stress response and the role of LisRK and CesRK in *Listeria monocytogenes*. *Microbiology* 158(Pt 4)**,** 963-974. doi: 10.1099/mic.0.055467-0.

an der Veen, S., van Schalkwijk, S., Molenaar, D., de Vos, W.M., Abee, T., and Wells-Bennik, M.H. (2010). The SOS response of *Listeria monocytogenes* is involved in stress resistavnce and mutagenesis. *Microbiology* 156(Pt 2)**,** 374-384. doi: 10.1099/mic.0.035196-0.

Rea, R., Hill, C., and Gahan, C.G. (2005). *Listeria monocytogenes* PerR mutants display a small-colony phenotype, increased sensitivity to hydrogen peroxide, and significantly reduced murine virulence. *Appl Environ Microbiol* 71(12)**,** 8314-8322. doi: 10.1128/AEM.71.12.8314-8322.2005.
