# Supplementary material for: Inactivation of lmo0946 (sif) induces the SOS response and MGEs mobilization and silences the general stress response and virulence program in Listeria monocytogenes
Source: Front Microbiol. 2024 Jan 4;14:1324062. doi: 10.3389/fmicb.2023.1324062 (PMC10794523; doi:10.3389/fmicb.2023.1324062)
Supplement: Supplementary file 10 [file Table_7.pdf]

Supplementary Table S7. Expression of MGE genes in *L. monocytogenes lmo0946**

Gene name	Gene symbol	MGE name [1]	$\log_2$ Fold Change	*P*adj[2]	Product [3]
lmo0113		**monocin (lma operon): *lmo0113 - lmo0129***	**0.98**	**8.38E-13**	similar to protein gp35 from Bacteriophage A118
lmo0114			**0.92**	**1.44E-11**	similar to putative repressor C1 from lactococcal bacteriophage Tuc2009
lmo0115	*lmaD*		**3.74**	**1.63E-57**	Listeria protein LmaD. associated with virulence
lmo0116	*lmaC*		**3.66**	**6.64E-60**	LmaC. associated with virulence in Listeria
lmo0117	*lmaB*		**4.61**	**5.6E-134**	Antigen B
lmo0118	*lmaA*		**4.52**	**4.3E-130**	Antigen A
lmo0119			**4.61**	**1.03E-65**	Hypothetical protein
lmo0120			**4.50**	**2.02E-84**	Hypothetical protein
lmo0121			**4.28**	**2.3E-109**	Phage tail length tape-measure protein
lmo0122			**4.23**	**1.6E-128**	Phage tail fiber
lmo0123			**4.15**	**1.3E-135**	Putative tail or base plate protein gp18 [Bacteriophage A118]
lmo0124			**3.96**	**9.22E-82**	Hypothetical protein
lmo0125			**4.26**	**5.7E-108**	Hypothetical protein
lmo0126			**4.32**	**4.82E-81**	Hypothetical protein
lmo0127			**4.24**	**8.1E-83**	Hypothetical protein
lmo0128			**3.99**	**5.77E-90**	Similar to phage-related protein
lmo0129			**4.12**	**2E-120**	N-acetylmuramoyl-L-alanine amidase
lmo1097		**ICELm1 (Tn916): *lmo1097 - lmo1115***	**13.71**	**4.63E-24**	Integrase. superantigen-encoding pathogenicity islands SaPI
lmo1098			0.04	NA	
lmo1099			0.04	NA	
lmo1100	*cadA*		**12.27**	**3.03E-19**	Cadmium resistance protein
lmo1101	*lspB*		**9.12**	**1.27E-10**	Hypothetical protein
lmo1102	*cadC*		**7.85**	**7.51E-08**	Cadmium efflux system accessory protein
lmo1103			0.15	NA	
lmo1104			0.22	0.249	
lmo1105			**6.36**	**1.24E-04**	Membrane protein. putative
lmo1106			**5.18**	**0.002**	Hypothetical protein
lmo1107			0.19	NA	
lmo1108			0.04	NA	
lmo1109			0.05	NA	
lmo1110			0.00	NA	
lmo1111			0.32	0.138	
lmo1112			**5.29**	**0.001**	Hypothetical protein
lmo1113			4.35	0.010	
lmo1114			0.46	0.064	
lmo1115			**4.96**	**0.002**	Similar to fibrinogen-binding protein
lmo2271		**A118: *lmo2271 - lmo2332***	**3.51**	**1.82E-42**	Hypothetical protein
lmo2272			0.20	0.657	
lmo2273			0.13	0.565	
lmo2274			0.62	0.012	
lmo2275			**0.73**	**2.97E-05**	Portein gp28 [Bacteriophage A118]
lmo2276			0.04	0.855	
lmo2277			0.50	0.013	
lmo2278	*lysA*		**3.09**	**4.3E-20**	L-alanoyl-D-glutamate peptidase
lmo2279			**2.78**	**4E-08**	Holin
lmo2280			**2.45**	**1.50E-04**	Protein gp23
lmo2281			0.04	NA	
lmo2282			**3.59**	**6.92E-16**	protein gp21
lmo2283			**2.84**	**3.26E-21**	Protein gp20
lmo2284			**3.13**	**1.65E-39**	Protein gp19

lmo2285		**3.51**	**3.8E-47**	Protein gp18
lmo2286		**2.75**	**1.46E-22**	Protein gp17
lmo2287		**3.20**	**3.91E-70**	Putative tape-measure
lmo2288		**3.74**	**6.22E-28**	Protein gp15
lmo2289		**4.35**	**4.71E-26**	Protein gp14
lmo2290		**3.24**	**1E-07**	Protein gp13
lmo2291		**3.68**	**2.36E-45**	Major tail shaft protein
lmo2292		**4.19**	**1.47E-40**	Protein gp11
lmo2293		**3.39**	**6.08E-45**	Protein gp10
lmo2294		**4.53**	**9.8E-35**	Protein gp9
lmo2295		**3.30**	**2.07E-42**	Protein gp8
lmo2296		**3.62**	**1.66E-94**	Phage capsid protein
lmo2297		**3.29**	**2.51E-35**	Putative scaffolding protein
lmo2298		**3.81**	**1.68E-91**	Protein gp4
lmo2299		**3.46**	**2.4E-106**	Putative portal protein
lmo2300		**3.33**	**9.31E-43**	Putative terminase large subunit from bacteriophage A118
lmo2301		**3.14**	**3.03E-21**	Terminase small subunit [Bacteriophage A118]
lmo2302		**0.81**	**3.24E-04**	Hypothetical protein
lmo2303		**3.06**	**4.53E-29**	Protein gp66
lmo2304		0.22	0.273	
lmo2305		**2.80**	**2.02E-23**	Hypothetical protein. Lmo2305 homolog [Bacteriophage A118]
lmo2306		**2.34**	**6.51E-10**	Hypothetical protein
lmo2307		0.55	0.241	
lmo2308		**2.34**	**7.28E-16**	Single-stranded DNA-binding protein (prophage associated)
lmo2309		0.89	0.074	
lmo2310		0.39	0.189	
lmo2311		**2.46**	**3.17E-06**	Hypothetical protein
lmo2312		**2.79**	**1.04E-06**	Conserved hypothetical protein
lmo2313		**3.75**	**6.7E-29**	Hypothetical protein. Lmo2313 homolog [Bacteriophage A118]
lmo2314		**3.88**	**0.003**	Hypothetical protein
lmo2315		**3.90**	**3.23E-41**	Protein gp51 [Bacteriophage A118]
lmo2316		**3.88**	**2.46E-50**	Methyltransferase
lmo2317		**3.35**	**4.99E-35**	Protein gp49. replication initiation [Bacteriophage A118]
lmo2318		**3.52**	**1.06E-23**	Putative recombination protein / Single-stranded DNA-binding protein
lmo2319		**3.50**	**1.29E-24**	Hypothetical protein
lmo2320		**2.88**	**2.24E-04**	Hypothetical protein
rli140		**3.14**	**2.66E-04**	
lmo2321		**4.35**	**2.42E-12**	Protein gp45 [Bacteriophage A118]
lmo2322		**3.64**	**1.26E-15**	gp44
lmo2323		**3.58**	**3.78E-38**	Protein gp43 [Bacteriophage A118]
lmo2324		**4.12**	**2.81E-59**	Phage antirepressor protein / Antirepressor [Bacteriophage A118]
lmo2325		**4.81**	**2.65E-21**	Hypothetical protein
lmo2326		**3.82**	**1.89E-11**	Protein gp41 [Bacteriophage A118]
lmo2327		**3.59**	**3.08E-22**	Hypothetical protein
lmo2328		**1.84**	**0.009554**	Similar to transcription regulator
lmo2329		0.03	0.887	
lmo2330		**0.49**	**0.004**	Similar to protein gp33 [Bacteriophage A118]
lmo2331		0.08	0.781	
int		**1.25**	**6.03E-16**	Integrase [Bacteriophage A118]
lmo0459	**IS3-I:**	0.40	0.034	
lmo0460	***lmo0459 - lmo463***	0.18	0.612	
lmo0461		-0.14	0.707	

lmo0462	-0.14	0.756
lmo0463	-0.02	0.962

[1] Information on MGE according to Kuenne et al.. 2013 (Kuenne C. Billion A. Mraheil MA. Strittmatter A. Daniel R. Goesmann A. Barbuddhe S. Hain T. Chakraborty T. Reassessment of the *Listeria monocytogenes* pan-genome reveals dynamic integration hotspots and mobile genetic elements as major components of the accessory genome. BMC Genomics. 2013. 22;14:47. doi: 10.1186/1471-2164-14-47)

[2] Log_2 expression levels with an adjusted p value of MGE genes in mutant *lmo0946** vs *Listeria monocytogenes* EGD-e from exponential phase of growth in BHI in 37 °C; In bold genes with upregulated expression (*P*adj < 0.01)

[3] Information on upregulated MGE genes from Listeriomics website (listeriomics.pasteur.fr)
