# Supplementary material for: Inactivation of lmo0946 (sif) induces the SOS response and MGEs mobilization and silences the general stress response and virulence program in Listeria monocytogenes
Source: Front Microbiol. 2024 Jan 4;14:1324062. doi: 10.3389/fmicb.2023.1324062 (PMC10794523; doi:10.3389/fmicb.2023.1324062)
Supplement: Supplementary file 11 [file Table_8.pdf]

Supplementary Table S8. Comparison of expression of SOS response genes in *L. monocytogenes lmo0946** to the expression observed in wild-type *L. monocytogenes* EGD-e strain after MMC treatment.

Gene name	Gene symbol	Description of product[1]	MMC treatment wt[2]	*lmo0946**[3]	*P*adj[3]
lmo0157		Predicted ATP-dependent helicase	1.61	**1.06**	**1.97E-21**
lmo0158		Predicted hydrolase	1.18	0.13	0.53
lmo1302	*lexA*	Transcription repressor of SOS response	1.74	**0.77**	**9.71E-09**
lmo1303	*yneA*	Similar to B. subtilis YneA protein	3.95	**1.91**	**0.001**
lmo1398	*recA*	Transcription activator of SOS response	2.58	**1.23**	**1.33E-38**
lmo1421	*bilEA*	Osmoprotectant transport system ATP-binding protein, bile resistance	1.13	-0.07	0.81
lmo1422	*bilEB*	Osmoprotectant transport system permease protein, bile resistance	1.21	0.09	0.74
lmo1574	*dnaE*	DNA polymerase III alpha subunit	1.37	**0.61**	**1.52E-10**
lmo1640		Hypothetical protein	1.90	**1.61**	**1.2E-12**
lmo1639		DNA-3-methyladenine glycosidase, base excision repair	2.24	**1.89**	**1.8E-40**
lmo1638		redicted peptidase	2.22	**1.62**	**1.08E-25**
lmo1759	*pcrA*	ATP-dependent DNA helicase	0.93	**0.50**	**1.49E-08**
lmo1758	*ligA*	NAD-dependent DNA ligase	0.65	**0.42**	**3.36E-05**
lmo1975	*dinB*	DNA polymerase IV	3.08	**2.29**	**6.85E-96**
lmo2222		Predicted DNA repair exonuclease	1.95	**1.54**	**1.8E-38**
lmo2221		Hypothetical protein	2.38	**1.53**	**2.93E-46**
lmo2220		Predicted exonuclease	1.52	**0.74**	**6.64E-18**
lmo2268	*addB*	Predicted ATP-dependent helicase	1.59	**1.05**	**4.96E-31**
lmo2267		Predicted ATP-dependent helicase	1.74	**0.87**	**2.69E-18**
lmo2266		Predicted hydrolase	1.56	0.44	0.12
lmo2265		Hypothetical protein	1.61	**1.32**	**5.05E-08**
lmo2264		Hypothetical protein	1.28	**0.81**	**2.98E-12**
lmo2271		Bacteriophage A118 protein	3.4	**3.51**	**1.82E-42**
lmo2332	*int*	Site-specific DNA recombinase, integrase (Bacteriophage A118)	1.58	**1.25**	**6.03E-16**
lmo2489	*uvrB*	Excinuclease ABC (subunit B)	2.76	**1.82**	**1.8E-34**
lmo2488	*uvrA*	Excinuclease ABC (subunit A)	2.63	**1.61**	**1.75E-58**
lmo2675	*umuD*	DNA polymerase V	2.93	**3.70**	**3E-128**
lmo2676	*umuC*	DNA polymerase V	2.14	**3.70**	**1.8E-172**
lmo2828		Predicted equivalent to the UmuD subunit of polymerase V from Gram-negative bacteria	4.56	**2.58**	**8.74E-17**

[1] Information from van der Veen et al., 2010

[2] Log_2 expression levels of genes from SOS regulon in the wild-type (Wt) strain after MMC treatment vs the wild-type untreated according to van der Veen et al., 2010 (van der Veen S., van Schalkwijk S., Molenaar D., de Vos W. M., Abee T., Wells-Bennik M. H. J. (2010). The SOS response of *Listeria monocytogenes* is involved in stress resistance and mutagenesis. Microbiology 156, 374-384. DOI 10.1099/mic.0.035196-0.)

[3] Log_2 expression levels of genes from SOS regulon in mutant *lmo0946** vs *Listeria monocytogenes* EGD-e from exponential phase of growth without stress factors; in bold genes with essentially changed expression (*P*adj < 0.01)
