# Supplementary material for: Inactivation of lmo0946 (sif) induces the SOS response and MGEs mobilization and silences the general stress response and virulence program in Listeria monocytogenes
Source: Front Microbiol. 2024 Jan 4;14:1324062. doi: 10.3389/fmicb.2023.1324062 (PMC10794523; doi:10.3389/fmicb.2023.1324062)
Supplement: Supplementary file 12 [file Table_9.pdf]

Supplementary Table S9. Expression of PrfA regulon in *L. monocytogenes lmo0946**

Gene Group[1]	Gene name[1]	Regulation[1]	*lmo0946**[2]	Padj[2]	Description of product[1]
**Group I** (positively regulated and preceeded by PrfA box; downregulated in presence of celobiose and upregulated in presence of charcoal)	***hly***	PrfA box	**-0.49**	**0.001**	listeriolysin O precursor
	mpl	PrfA box	**-0.66**	**0.019**	Zinc metalloproteinase precursor
	actA	PrfA box	**-0.52**	**0.014**	actin-assembly inducing protein precursor
	plcB	PrfA box	-0.09	0.756	
	plcA	PrfA box	-0.46	0.126	
	prfA	PrfA box; SigB	**1.36**	**7.79E-10**	Listeriolysin O positive regulatory protein
	inlA	PrfA box	**-1.30**	**3.02E-05**	Internalin A
	inlB	PrfA box	**-1.09**	**4.93E-04**	Internalin B
	inlC	PrfA box	0.40	0.167	
	uhpT	PrfA box	-0.45	0.262	
	lmo2219	PrfA box	-0.18	0.36	
	lmo0788	PrfA box	**-2.28**	**1.30E-38**	Unknown
**Group II** (negatively regulated by PrfA)	*lmo0178*		-0.42	0.149	
	lmo0179		-0.05	0.860	
	lmo0180		-0.16	0.527	
	lmo0181		0.19	0.431	
	lmo0182		0.23	0.304	
	lmo0183		0.31	0.058	
	lmo0184		-0.02	0.931	
	lmo0278	PrfA box	**-0.83**	**1.49E-05**	Unknown, similar to sugar ABC transporter, ATP-binding protein
**Group III** (positively regulated by PrfA; upregulated in presence of celobiose and downregulated in presence of charcoal)	***lmo0596***	PrfA box; SigB	**-1.09**	**0.0367**	Unknown, similar to unknown proteins
	lmo2067	PrfA box; SigB	**-2.23**	**2.74E-08**	Unknown, similar to conjugated bile acid hydrolase
	opuCA	SigB	-0.51	0.268	
	opuCB		-0.49	0.285	
	opuCC		-0.35	0.430	
	opuCD		-0.52	0.258	
	lmo1602	SigB	**-0.71**	**1.12E-08**	similar to general stress protein
	lmo1601		**-0.66**	**2.29E-08**	similar to general stress protein
	lmo2748	SigB	**-1.25**	**8.12E-04**	Unknown, similar to *B. subtilis* stress protein YdaG
	lmo2230	SigB	-0.53	0.237	
	lmo2231		-0.39	0.364	
	lmo0913	SigB	-0.89	0.094	
	lmo0669	SigB	-0.52	0.153	
	lmo0670		**-0.90**	**0.023**	Unknown
	lmo2573		**-1.34**	**0.013**	Unknown, similar to zinc-binding dehydrogenase
	lmo2572		**-1.13**	**0.004**	Unknown, similar to Chain A, Dihydrofolate Reductase
	lmo2571		**-1.38**	**6.82E-05**	Unknown, similar to nicotinamidase
	lmo2570		-0.32	0.471	
	lmo2695	SigB	-0.82	0.095	
	lmo2696		-0.47	0.297	
	lmo2697		-0.39	0.391	
	lmo1694	SigB	-0.86	0.101	
	lmo0539	SigB	-0.66	0.160	
	lmo0784	SigB	**-1.79**	**1.07E-11**	Unknown, similar to mannose-specific phosphotransferase system (PTS) component IIA
	lmo0783		**-1.43**	**2.39E-05**	Unknown, similar to mannose-specific phosphotransferase system (PTS) component IIB
	lmo0782		**-1.00**	**0.005**	Unknown, similar to mannose-specific phosphotransferase system (PTS) component IIC
	lmo0781		-0.49	0.278	

lmo0602	SigB	**-1.56**	**4.05E-06**	Unknown, weakly similar to transcription regulator
lmo2391	SigB	**-1.39**	**4.31E-05**	Unknown, conserved hypothetical protein similar to *B. subtilis* YhfK protein
lmo0043		**-1.84**	**4.79E-07**	Unknown, similar to arginine deiminase
lmo0133		**-2.35**	**4.34E-16**	Unknown, similar to *E. coli* YjdI protein
lmo0134		**-2.40**	**8.36E-16**	Unknown, similar to *E. coli* YjdJ protein
lmo0937	SigB	**-2.37**	**3.70E-15**	Unknown
lmo0994	SigB	-0.65	0.174	
lmo0794	SigB	**-1.05**	**0.004**	Unknown, similar to *B. subtilis* YwnB protein
lmo0654		**-2.68**	**7.63E-33**	Unknown
lmo2213	SigB	**-1.47**	**0.025**	Unknown, similar to unknown protein
lmo2673		**-1.43**	**0.016**	Unknown, conserved hypothetical protein
sepA	SigB	**-1.11**	**0.047**	Unknown
lmo0796		**-1.02**	**1.29E-05**	Unknown, conserved hypothetical protein
lmo1261	SigB	**-0.91**	**0.010**	Unknown
lmo0439	SigB	**-1.35**	**0.018**	Unknown, weakly similar to a module of peptide synthetase
lmo0953		**-2.07**	**6.20E-14**	Unknown
lmo0019		**-1.09**	**0.017**	Unknown
lmo0555		-0.54	0.058	
lmo0676		0.45	0.215	
inlH		-0.87	0.102	
lmo0169		**-1.41**	**1.59E-04**	Unknown, similar to a glucose uptake protein
lmo0170		**-0.71**	**0.020**	Unknown
lmo0242		-0.26	0.167	
rsbV	SigB	**-1.05**	**1.47E-06**	anti-anti-sigma factor (antagonist of RsbW)
lmo0641	SigB	**1.14**	**2.59E-07**	Unknown, similar to heavy metal-transporting ATPase
galE		**-0.34**	**0.006**	UDP-glucose 4-epimerase

[1] Information from Milohanic et al., 2003 (Milohanic, E., Glaser, P., Coppee, J.Y., Frangeul, L., Vega, Y., Vazquez-Boland, J.A., et al. (2003). Transcriptome analysis of *Listeria monocytogenes* identifies three groups of genes differently regulated by PrfA. Mol Microbiol 47(6), 1613-1625.); genes in operons are boxed

[2] Log_2 expression levels with an adjusted p value of genes from PrfA regulon in mutant *lmo0946** vs *Listeria monocytogenes* EGD-e from exponential phase of growth without stress factors;

In red genes with upregulated expression (*P*adj < 0.01); in dark blue genes with downregulated expression (*P*adj < 0.01); in light blue genes with downregulated expression (*P*adj < 0.05);
