# Supplementary material for: Inactivation of lmo0946 (sif) induces the SOS response and MGEs mobilization and silences the general stress response and virulence program in Listeria monocytogenes
Source: Front Microbiol. 2024 Jan 4;14:1324062. doi: 10.3389/fmicb.2023.1324062 (PMC10794523; doi:10.3389/fmicb.2023.1324062)
Supplement: Supplementary file 13 [file Table_10.pdf]

Supplementary Table S10. Expression levels of genes from *sigB* operon in mutant *lmo0946** vs *Listeria monocytogenes* EGD-e from exponential phase of growth in 37 °C.

Gene name	Gene symbol	log_2 Fold Change	*P*adj	Product [1]
lmo0889	*RsbR*	0.039	0.758	RsbR, positive regulator of sigma-B
lmo0890	*rsbS*	0.058	0.728	RsbS, negative regulator of sigma-B
lmo0891	*rsbT*	0.228	0.103	Anti-sigma B factor RsbT
lmo0892	*rsbU*	-0.061	0.72	Serine phosphatase RsbU, regulator of sigma subunit
lmo0893	*rsbV*	-1.048	1.47E-06	anti-anti-sigma factor (antagonist of RsbW)
lmo0894	*rsbW*	-0.478	0.002	serine-protein kinase RsbW
lmo0895	*sigB*	-0.355	0.138	RNA polymerase sigma factor SigB
lmo0896	*rsbX*	-0.281	0.193	indirect negative regulation of sigma B dependant gene expression (serine phosphatase)

[1] Information from Listeriomics website (listeriomics.pasteur.fr)
Genes preceded by SigB promoter located within the *sigB* operon are boxed.
